# Barriers towards HPV Vaccinations for Boys and Young Men: A Narrative Review

**DOI:** 10.3390/v13081644

**Published:** 2021-08-19

**Authors:** Maria Grandahl, Tryggve Nevéus

**Affiliations:** Department of Women’s and Children’s Health, Uppsala University, 751 85 Uppsala, Sweden; tryggve.neveus@kbh.uu.se

**Keywords:** attitudes, beliefs, boys, human papillomavirus, HPV vaccination, immunization programs, men, review

## Abstract

Background: HPV vaccination of both girls and boys can protect against infection and eliminate the risk for HPV-associated cancer. Due to a common misconception that the virus only poses risks to women, vaccine coverage is suboptimal among men in many countries. It is urgent to identify barriers to vaccination of boys and men. Methods: We conducted a narrative review of publications examining attitudes and beliefs regarding HPV vaccination for boys and young men. The electronic databases searched were PubMed, PsychInfo and Scopus (December 2020; last update July 2021). A total of 103 original articles were included in the final analysis. Results: The central barriers against vaccination of boys and men are: (1) lack of knowledge, (2) vaccine hesitancy in general, (3) lack of recommendation from and/or discussions with healthcare providers, (4) cost and logistics, and (5) the idea that HPV vaccination may promote promiscuity. Men who have sex with men and families belonging to ethnic minorities express a need for information tailored to their situation. Conclusions: Boys should be included in national immunization programs and men should also be offered catch-up vaccinations. Future studies should focus on addressing vaccine hesitancy and developing interventions to promote pan-gender HPV vaccination.

## 1. Introduction

Human papillomavirus (HPV) infection is a substantial contributor to the global cancer burden. The virus is transmitted through sexual contact and infection is extremely common both in women and men; the estimated lifetime probability of being infected is above 80% [1]. High-risk HPV types can cause precancerous lesions, which, in turn, may progress to cervical cancer—the second most common malignancy among women aged 15–44 years. The virus is also a major cause of cancer in the head and neck region, anus, vulva, vagina and penis. Thus, men are also at risk [2,3].

Vaccine hesitancy in society is a growing challenge and was in 2019 listed by the World Health Organization (WHO) as one of the top 10 global public health threats. The stated WHO goal is to achieve a worldwide HPV vaccine coverage of ≥90%. However, misconceptions and fake news can disrupt national vaccination programs [4,5,6].

National HPV vaccination programs are implemented in at least 107 countries worldwide [7]. Australia, in 2013, was the first country to also include boys. Several European countries, as well as, for instance, Argentina, Canada, Israel and New Zealand, have recently followed Australia’s example. About one third of these countries (33/107) have included boys in the national vaccination program. Globally, in 2019 about 4% of boys had received the full course of the vaccine compared to 15% of girls [7]. The vaccine is highly efficient and safe and has resulted in a radical reduction of cervical lesions and condylomas. To achieve the best protection, it needs to be given to HPV-naïve persons, i.e., prior to sexual debut. Unfortunately, vaccine uptake in most countries is suboptimal and the coverage varies both between and within countries [7,8]. Pan-gender vaccination is the only way to have a chance of eradicating HPV infections and HPV-associated cancer [9]. Thus, HPV and HPV vaccination needs to be seen not as a women’s problem but a responsibility for everyone. Therefore, it is urgent to vaccinate boys and men. This would be beneficial for the individual as well as public health [10].

The aim of this review is to provide a concise update on the latest progress made about HPV vaccination in boys and men, and the barriers against vaccine coverage of this half of humanity.

## 2. Materials and Methods

This narrative review was performed in line with Onwuegbuzie and Frels (2016), and Polit and Beck (2016) [11,12]. A comprehensive search strategy was developed in consultation with a senior librarian at Uppsala University. In addition, both authors (MG and TN) individually searched articles. We used the following keywords: HPV vaccination and boys and young men, attitudes, beliefs and barriers. The electronic databases searched were PubMed, PsycInfo and Scopus (date of initial search December 2020; last update July 2021). Furthermore, reference lists of all identified studies were manually screened. Original studies that included HPV vaccination, boys, men and attitudes, beliefs, views and barriers written in English and published in 2010–2021 were eligible. A total of 103 studies conducted in North America, South America, Asia, Europe and Oceania on the topic of HPV vaccination and boys/men, with a special focus on attitudes and perceived barriers, were included in our final analysis.

Based on the material found, and our personal expertise, we conducted a narrative review [11,12] to provide an overview for non-experts who are involved in the care of men with HPV-related cancers.

## 3. Findings

### 3.1. The Perspective of the Relevant Key Groups

#### 3.1.1. The Perspective of the Boys

Boys have, in general, lower knowledge about HPV compared with girls [13].

There is a broad variation in boys’ opinions about HPV vaccination, and whether they intend to be vaccinated or not. Only 29% were willing to be vaccinated among boys in the USA and 37% in France, while a Swedish study found that boys seem to be in favor of being vaccinated [14,15,16].

According to a Dutch study, fear of unknown side effects is a cause behind the boys’ vaccine hesitancy [17]. Unsurprisingly, knowledge and awareness of cancer correlates with the intention to vaccinate [13].

Schoolboys believe that the school health service is an adequate arena for information about HPV and HPV vaccination. Information from healthcare providers increases the chances that adolescents in low-income families will become vaccinated; adolescents who recalled that their provider had given them written information were more likely to complete the vaccine series [18]. Boys also consider vaccination against HPV a matter of equal rights [16].

#### 3.1.2. The Parental Perspective

The parental perspective mirrors that of the boys themselves: knowledge is poor [19,20,21,22,23,24] and there is a lingering misconception that this is a female problem [21]. Many parents who had accepted the vaccine for their daughters were not aware of the recommendation or the benefits for boys [25,26].

The intention among parents to have their sons vaccinated varies considerably between countries and sociodemographic groups. Parents in Brazil seem to be in favor with an acceptance of 86% [25], whereas parts of the USA (43%) and France (38%) present much lower figures [14,15]. Mothers are in favor of vaccinating their sons [27] but there may be a discrepancy between their stated belief that vaccination is important and their actual intention to have their sons vaccinated [28]. Their intention may not always mean that the child in the end becomes vaccinated [29]. Thus, boys are lost in every step from vaccine-favorable parental opinions to actual vaccination. Fathers have low knowledge and awareness of HPV. Nonetheless, they are usually in favor of vaccinating their sons [30], although perhaps less so than the mothers [31,32]. In general, parents of boys seem to be unsure about HPV vaccination to a greater extent compared to parents of daughters [33].

As was the case with the boys themselves, increased parental knowledge predicts acceptance of the vaccine [18,34]. Several studies have found that parents who had discussed sexual health with their sons were more likely to vaccinate [35,36]. On the other hand, many parents were afraid that the HPV vaccine would promote promiscuity [37], and fathers especially were more likely to link risk to female promiscuity [38]. Furthermore, parents believed that vaccination was not needed since the child was too young and was not sexually active [21,37,39,40]. However, it should be noted that parents tend to overestimate the age of sexual debut for their adolescent children [41]. Further reasons not to vaccinate the sons were the belief that the vaccine is harmful and/or gives limited benefit [42], fear of unknown side-effects [17,33,40] and vaccine hesitancy in general [15,42]. Cost is also a significant barrier, especially for economically underprivileged parents [27,43].

Finally, perceived support from others in the decision is a facilitator for vaccine completion [27], and recommendations from healthcare providers may be the single most important factor for HPV vaccination. A US study among Hispanics found that initiation was higher among sons whose parents had received a recommendation to vaccinate from a healthcare provider compared with those whose parents had not [44].

#### 3.1.3. Young Men’s Perspective

Men are much less HPV vaccinated than women [45,46,47]. Although knowledge about HPV is low among young people, in general [48,49,50] studies indicate that men have even lower knowledge than women [51,52]. They may be aware of cervical cancer but they tend to have inadequate knowledge about HPV and HPV vaccination [37,53,54] and they perceive their own susceptibility to be affected by the virus to be low [55].

As with boys and parents, there are great differences between countries/regions, as an example: the willingness to vaccinate is high in California but low in Hong Kong [54,56].

Concerns that vaccination would lead to promiscuous behavior as well as the perception that one will only have one partner, i.e., not be sexually active until marriage, are other reasons for unwillingness to vaccinate against HPV [37]. Arguably, this concern may be given some support by the report that young men reporting oral sex behaviors indicate vaccine intent [57], and students who had had sexual intercourse were significantly more likely to support HPV vaccination [58] for both girls and boys [59].

As usual, knowledge—especially knowledge regarding men’s risk to be affected by HPV—increases vaccine acceptance [56,60]. Furthermore, there is a correlation between young men’s perceived self-efficacy in health practices and HPV vaccination intentions [60].

#### 3.1.4. Healthcare Providers’ (HCPs) Perspective

Obviously, HCPs play a key role in the success of HPV vaccination programs, since the attitudes and confidence of these professionals affect vaccine acceptance [61,62,63,64]. Population based studies have found that school nurses are generally in favor of vaccinating boys against HPV [65,66]. Although HCPs are generally in favor of including boys in national vaccination programs [67,68], since this is as a way to reduce social inequalities [65,66,69], they routinely recommend vaccination to girls more often than to boys [70,71,72].

HCPs face challenges such as parental vaccine hesitancy and lack of time to provide adequate information to parents and children [65,66,70]. They request more information about HPV [65,66,67] and experience discomfort when discussing the link between HPV and sexual behavior, especially when explaining oral and anal HPV transmission [73]. Finally, even this group are concerned that the vaccine may decrease condom use [74].

#### 3.1.5. The Perspective of Men Who Have Sex with Men (MSM)

There is a broad variation in knowledge and awareness about HPV and HPV vaccination among MSM. An Irish study on young MSM found that only half (54.9%) of them were aware of HPV [75], and similar results were found in an Italian study in which 54.6% had heard about HPV infection and fewer, 42%, were aware of HPV vaccination [76]. A Chinese study among MSM found even lower knowledge about HPV and HPV vaccination (with a mean score of 1.9 points (range 0–11 points)) [77]. Low risk-perception and awareness of the virus and HPV-related cancer was also found among African-American MSM [78]. In contrast, other US studies show that almost all participants (93%) had heard of HPV, although they were often not aware of the link between infection and cancer [79,80].

Despite low awareness among many men [81,82], MSM tend to know more about the virus than other men [83,84,85], and they have in general a more favorable attitude towards HPV vaccination [76,79,83,84,86,87].

In spite of these favorable beliefs and attitudes, only a minority have discussed the vaccine with HCPs [75]. MSM also report feelings of stigma and emphasize that HCPs need better knowledge about gay, bisexual and queer individuals [86]. Nonetheless, MSM have trust in healthcare, request more information [86], and the information given is an important facilitator for vaccination in this group [82,88].

As in the other groups, MSM consider the cost a barrier against vaccination [79,89], but the stigma of openly discussing same-sex experiences with HCPs is a barrier peculiar to this group [81].

### 3.2. The Impact of Cultural, Sociodemographic and Religious Factors

There are differences in attitudes and beliefs about HPV vaccination for boys and young men between countries and cultural groups. The acceptability of vaccinating young boys against HPV is high (83%) in Brazil [25] while it is low (23.3%) among male students in Hong Kong [56]. However, the bulk of the research, so far, is undertaken in the USA and the number of studies about HPV vaccination for boys and men undertaken in low- or middle-income countries is low [90].

Cultural norms and religious and ethnic customs definitively play a role as determinants of vaccine acceptance [91].

Several barriers are found among Latinos in the USA who have low knowledge about HPV, have low confidence in vaccine efficacy, and perceive low vaccine benefits compared to other groups [23,24,92]. Furthermore, cost is a main barrier for vaccination in this group [27]. A similar situation is described among Somali-American men [93]. Traditional patriarchal gender roles may have an impact here [92], and there may be a cultural taboo against discussing sexuality and sexual health, as well as a perception that HPV vaccination is for females and the previously mentioned belief that the vaccine is associated with promiscuity are barriers due to cultural and ethnic factors [53,92].

Although minorities are often vaccinated to a lower extent, there are exceptions. For instance, in the Arabic community in Israel, boys and young men have high vaccine uptake [85,94]. Also, sociodemographic disadvantage is an important factor which is difficult to disentangle from ethnicity. In general, high parental educational level is predictive for vaccine completion [18], but the findings are inconsistent [28].

Importantly, minority groups request targeted information about HPV and HPV vaccination adjusted to their cultural and ethnic group [39].

Finally, religion has an impact on beliefs and knowledge about HPV vaccination, and the decision-making process to vaccinate or not [95,96]. One study among young students in a Christian University in the US showed low knowledge of the virus and lower vaccine acceptance rates compared to the national average [37]. Furthermore, students with a low religious commitment were more likely to be vaccinated than students who were highly committed to their faith [37]. Christian parents often believe that their sons shall stay chaste until marriage and are concerned that the vaccine will lead to promiscuity [37]. In a similar vein, Muslim Somali adolescents perceive low risk due to religiously sanctioned sexual abstinence [97].

### 3.3. Specific Barriers to Human Papillomavirus (HPV) Vaccination

#### 3.3.1. Low Knowledge

As should be clear from the above sections, low HPV and HPV vaccine knowledge is a major barrier against vaccination. In particular, the link between HPV and oral and anal cancer is not sufficiently well known [80,87]. Knowledge has been found to be an independent predictor of vaccine acceptance [60]. Nonetheless, some studies describe young men with low knowledge who are in favor of HPV vaccination anyway [16,54].

#### 3.3.2. Ideas about Sexual Promiscuity

The perception that HPV vaccination is associated with increased risk of promiscuity is widespread among both parents, sons, and youth in general [37,98]. This idea may be especially common among minorities such as Hispanic fathers in the US [38] and similar findings are reported in studies among young men in Hong Kong [53,98].

This assumption is not supported by science. HPV-vaccinated Swedish girls in the catch-up program had less sexual risk-taking compared with girls who were not vaccinated [99]. Sexual risk-taking among Hungarian adolescents is not associated with increased HPV knowledge [100].

#### 3.3.3. Fear of Side-Effects

Fear of side-effects is a common barrier against parental acceptance of HPV vaccination [33,101]. We have here a spectrum of opinions from parents who are opposed to all vaccinations and/or have low trust in the healthcare system [25,33] to parents who are hesitant and undecided. The former group will continue to oppose vaccinations regardless of information given, whereas the latter might change their mind over time [34].

#### 3.3.4. Cost and Logistics

Obviously, to have to pay for the vaccine is an essential barrier [102] especially among those with low socioeconomic status [27,43,101]. A related problem is that the vaccine may be perceived to be hard to access. A US study among Haitian and African-American parents of adolescent sons found that although 64–79% of the included parents intended to vaccinate their adolescent sons, only about 20% had received the vaccine one year later [103].

An important and related factor is whether the vaccine is included in nationwide school-based vaccination programs, or if it is up to the parents or the young man himself to take the initiative [104]. In the former situation uptake will be high and in the latter case vaccine coverage will be substantially lower [105]. Several studies illustrate this situation by showing that recommendation from the family doctor is a crucial factor [44,106,107].

### 3.4. The Role of Knowledge and Knowledge Dissemination

As has been highlighted from several perspectives in this review, knowledge is crucial. The more one knows about HPV and the HPV vaccine, the greater the likelihood is that one will become vaccinated or have one’s sons vaccinated [106]. But how is this knowledge to be disseminated?

Mass media can have a positive effect and act as a vector for vaccine awareness [43,108] but a Canadian study showed that newspaper articles failed to mention the vaccine’s approval for males, and tended to report HPV’s relation to cervical cancer to the exclusion of other HPV-associated cancers [109].

Healthcare, and especially HCPs, play an undisputed role in informing about HPV and addressing misconceptions and unfounded fear [39,63,108,110,111,112]. This source is more influential in a parent’s decision compared to TV and other sources [113]. Thus, HCPs’ recommendations are clear motivators for parents’ decision to vaccinate their sons [106,107].

Only a handful of researchers have addressed the question of how to best disseminate knowledge and influence attitudes among adolescents of all sexes. There have been a few prospective school-based information interventions [49,50,114,115]. Educational school-based interventions can increase adolescents’ awareness and knowledge about HPV prevention and increase vaccination rates [115]. Furthermore, promising results and increased vaccination rates are found among young students attending university health services in a large university in the US. This student-directed informational intervention included recommendations from HCPs and effectively increased HPV vaccination rates [116].

## 4. Discussion

Vaccination against HPV may lead to the eradication not only of cervical cancer in women but also of cancers in the head and neck region, anal cancers and genital tumors in both men and women. But to achieve this goal vaccination needs to be given to boys as well, to *all* boys and before sexual debut. As should be clear from this review we are still a long way from reaching this goal.

Vaccine coverage of boys, not just girls, has been included in nationwide school-based vaccination programs across the globe, but uptake is patchy, knowledge is deficient and misconceptions linger. Central misconceptions that lead to diminished vaccine acceptancy are the following:

(1) HPV vaccination is only relevant for girls and women;
(2) HPV vaccination leads to increased promiscuity or sexual risk-taking;
(3) HPV vaccination is unsafe.

It is not surprising that parents of boys are more unsure about HPV vaccination compared to parents of daughters [33]. For a long time, the vaccine only targeted girls and women. This is hopefully gradually changing but will take time.

The nature of HPV transmission poses a challenge to parents that other infections do not: they have to discuss sexual health behavior with their sons. The parental opinion that HPV vaccination should be delayed since (they believe) their child is not sexually active [101] may partly reflect this unease. But we believe that a school-based HPV vaccination could have positive consequences by encouraging open discussions in the family.

Vaccine hesitancy is one of the greatest health challenges facing the world today [117]. This is a multifaceted problem related to lack of trust in authorities, lack of scientific literacy and biased or uninformed media reporting. It is above all a question of information. But the majority of parents who are concerned about vaccine risks are not stuck in antiscientific conspiracy theories: they can be reached and may listen to the evidence.

The role of HCPs in the success of national HPV immunization cannot be overstated [62,66,118,119,120]. A recurring theme in recent studies is that HCPs constitute the most trusted source of HPV information [44,106,121,122,123] and recommendations from physicians and nurses may often be the central factor deciding that a boy or a young man will become vaccinated. Thus, HCPs need sufficient knowledge about HPV-related cancer and even more, they need self-efficacy to address questions asked and concerns [66,70]. One way to strengthen HCPs’ self-efficacy is by providing continuous education and training about HPV and HPV vaccine.

School-based HPV vaccination programs are linked to high vaccine coverage and contribute to sociodemographic equality [69,124,125,126]. If we are to reach all boys—and all boys need to be reached—then the school is the best arena. Not all boys have a family physician but nearly all boys go to school. If HPV vaccination is left to the parents’ own initiative the economic and logistic challenges will ensure that many boys, especially among underprivileged groups, will not become protected.

Besides, school-based vaccination is an opportunity for HCPs to inform children about the virus and discuss sexual health in general, before the children have become sexually active. After all, children have the right to be informed about actions that can promote their future health. This is also in line with the Convention on the Rights of the Child [127].

So what can be done? Boys and young men seem to be in favor of being vaccinated against HPV [14,16,52,76,79,83,84,86,87,120]. One way to increase the global uptake is to offer catch-up vaccinations preferably in the school health system [126], at youth health clinics and in health facilities at colleges/universities. Furthermore, to offer catch-up vaccinations to boys and young men whose parents previously declined the vaccine, and not the least, to prevent future HPV-related cancer among high-risk groups such as MSM and transgender individuals would balance health inequalities and let the young men make informed decisions about prevention against future HPV-related diseases. Interventions targeting adolescents and university students show promising results [115,116]. School-based interventions can increase motivation to vaccinate and eradicate gender differences [13,115]. There is a need for interventions developed together with the stakeholders, i.e., HCPs, parents and the children themselves, both boys and girls (regardless of sexual identity). The school nurse and family physician should be encouraged to provide scientifically correct information about the virus, its transmission and its long-term risks. They should not shy away from non-judgmental discussions about sexual habits and the need to protect not just the individual but also their future sexual partners. They should be provided with information material that acknowledges diversity and addresses the differing situation of diverse ethnic minorities, cultural groups and sexual orientations.

This narrative review provides a concise update on the latest progress made about HPV vaccination in boys and men, and the barriers against them becoming vaccinated. Although the included studies provide a broad spectrum of the stakeholders’ perspective—i.e., boys and their parents, men, MSM and HCPs—and were undertaken in different countries and sociodemographic areas, it was impossible to include all relevant studies. We still believe that the crucial points have been made.

## 5. Conclusions

Much needs to be done if full HPV vaccination of all boys and young men is to be reached. The main barrier is a lack of knowledge and misconceptions regarding the vaccine’s safety and effectiveness. However, most parents, boys and young men will accept vaccination if they are given reliable information, and also have the opportunity to discuss HPV with healthcare providers. The goal can be reached.

## Data Availability

Not applicable.
